# Maternal supplementation with balanced fatty acid fat powder enhances sow reproductive performance and offspring intestinal health by modulating mitochondrial fusion and cell apoptosis

**DOI:** 10.3389/fnut.2025.1690257

**Published:** 2025-12-02

**Authors:** Kan Xiao, Yanbing Zhang, Minfang Zhang, Jiahao Liu, Junjie Guo, Shiwei Zhao, Xiao Xu, Shaokui Chen, Yulan Liu

**Affiliations:** 1 Hubei Key Laboratory of Animal Nutrition and Feed Science, Wuhan Polytechnic University, Wuhan, China; 2 Shandong Zhongmu Feed Technology Co., Ltd., Binzhou, Shandong, China; 3 Shandong Crelipids Biotechnology Co., Ltd., Binzhou, Shandong, China

**Keywords:** balanced fatty acids, intestinal health, mitochondrial fusion, cell apoptosis, pigs

## Abstract

**Introduction:**

Fatty acids play a critical role in meeting energy demands and maintaining intestinal health of pigs. Maternal dietary fatty acid composition may influence offspring growth and intestinal integrity. This study aimed to investigate the effects of supplementing sow diet with a balanced fatty acid fat powder (BFAFP) during late gestation and lactation on reproductive performance and offspring intestinal health, and to explore the underlying regulatory mechanisms.

**Methods:**

In Exp. 1, a total of 24 multiparous sows (d 90 of gestation) were divided into two groups including a control diet (U:S ratio: 5.08; n-6/n-3 PUFA ratio: 7.72) supplemented with 2% soybean oil and an experimental diet containing 2% BFAFP (U:S ratio: 2.98; n-6/n-3 PUFA ratio: 3.13) which was composed of fish oil, flaxseed oil, red palm oil, coconut oil, soybean oil, and lysophospholipids. At farrowing, sow blood and colostrum were collected. In Exp. 2, at d 21 of lactation, twenty-four suckling piglets were chosen and used in a 2 × 2 factorial arrangement and the main factors including diet (maternal supplementation of soybean oil or BFAFP) and lipopolysaccharide (LPS) challenge (saline or 100 μg/kg BW LPS injection). At 4 h post LPS injection, suckling pigs were killed for intestinal tissue sample collection.

**Results:**

Compared with soybean oil, maternal supplementation with BFAFP not only significantly enhanced the number of weaned piglets, weaning survival rate, and milk yield (*P* < 0.05), but also showed trends for increasing total litter weight at weaning (*P* = 0.073), total litter weight gain (*P* = 0.058), and individual piglet daily gain (*P* = 0.074). Maternal BFAFP supplementation increased the concentrations of lactose, protein and non-fat solids as well as IgA and IgM concentrations in colostrum (*P* < 0.05). Moreover, maternal BFAFP supplementation significantly increased jejunal villus height post-LPS challenge in piglets (*P* < 0.05). Maternal BFAFP supplementation also reduced jejunal mRNA expression of tumor necrosis factor α, and interleukin-1 β and mitigated LPS-induced downregulation of claudin-1 mRNA (*P* < 0.05). Moreover, Maternal BFAFP supplementation upregulated jejunal mRNA and protein expression of mitofusin-1 and mitofusin-2 after LPS challenge (*P* < 0.05). Finally, maternal BFAFP suppressed jejunal mRNA expression of caspase-3 and caspase-9 in piglets after LPS challenge (*P* < 0.05).

**Discussion:**

Maternal BFAFP supplementation enhanced sow reproductive performance and offspring growth while protecting offspring against LPS-induced intestinal damage, likely through promoting mitochondrial fusion and attenuating inflammatory response and cell apoptosis.

## 1 Introduction

Sow reproductive performance is a critical determinant of swine production efficiency, with maternal nutritional status significantly influencing offspring health and development (1). During the pre-weaning period, porcine neonates rely exclusively on sow milk for nutrition. Both colostrum intake and sustained milk consumption are essential for establishing optimal intestinal microbiota and developing mucosal immunity in piglets (2). Notably, the nutritional composition of sow milk is profoundly influenced by dietary formulations during lactation (2).

Fatty acids are a key class of nutrients that play vital roles in swine reproduction, growth, and health (3). These nutrients are directly transferred from sow to offspring through milk, supporting neonatal development (2). The fatty acid profile of maternal diet directly determines the fatty acid composition of milk (4). Different fatty acids exhibit distinct biological functions, affecting mucosal immune responses, epithelial barrier function, oxidative stress regulation, and inflammatory modulation (3). Long-chain omega-3 fatty acids, particularly eicosapentaenoic acid (EPA) and docosahexaenoic acid (DHA), have demonstrated protective effects against deoxynivalenol-induced intestinal cell injury and enhanced barrier function in porcine intestinal epithelial cells (5). In contrast, n-6 polyunsaturated fatty acids (PUFA) such as arachidonic acid typically exhibit pro-inflammatory properties (6). Emerging evidence suggests that maternal n-6:n-3 PUFA ratio significantly influences colostrum composition in sows and serum fatty acid profiles in piglets (7). Maintaining the dietary n-6:n-3 PUFA ratio within the range of 1:1 to 5:1 enhanced fatty acid absorption and amino acid utilization in growing-finishing pigs, while also contributing to improved lipid metabolism and inflammatory regulation (8). Furthermore, Wang et al. reported that the apparent and standardized ileal digestibility of fats and fatty acids increased linearly with increasing unsaturation-to-saturation (U:S) ratios in growing pigs, with optimal utilization efficiency observed at a U:S ratio of 4.14 and a minimum effective ratio of 2.91 (9). In animal production, oils are commonly used to supply fatty acids due to their rich and varied profiles. However, a single oil source rarely provides an ideal balance. Therefore, strategic blending is necessary to achieve target ratios. These balanced ratios are directly linked to key outcomes such as improved animal growth, enhanced product quality (7–9). Despite these advances, the effects of maternal balanced fatty acid fat powder (BFAFP) with appropriate n-6:n-3 PUFA and U:S ratios on offspring growth performance and intestinal health remain largely unexplored.

Mitochondria, as highly dynamic organelles with double membranes, play pivotal roles in cellular function (10). Maintaining mitochondrial abundance, integrity, and homeostasis is crucial for cellular function (11). Mitochondrial dynamics, governed by continuous fusion and fission processes, determine organelle morphology and functionality (12, 13). Mitochondrial fusion, mediated by proteins such as optic atrophy 1 (OPA1), mitofusin-1 (MFN1) and mitofusin-2 (MFN2) (14), has been implicated in intestinal health (15). Bao et al. (16) reported reduced OPA1 expression in the intestinal epithelial cells of patients with inflammatory bowel disease and showed that gut-specific OPA1 deficiency in mice resulted in spontaneous intestinal inflammation and cell death. However, no studies have investigated the effects of maternal appropriate n-6:n-3 PUFA and U:S ratio on mitochondrial fusion in offspring piglet intestines.

Therefore, this study aimed to evaluate the effects of BFAFP supplementation on sow reproductive performance, and to determine whether maternal BFAFP supplementation conferred protective effects on piglet intestinal health through modulation of mitochondrial fusion and cell apoptosis.

## 2 Materials and methods

### 2.1 Animals, diets, management and sample collection

All animal procedures were approved by the Animal Care and Use Committee of Wuhan Polytechnic University (Approval No. WPU202304005) and conducted in compliance with institutional guidelines for the care and use of laboratory animals. The trial was performed at Hubei Longwang Livestock Co., Ltd. (Jingmen, Hubei Province, China).

In Exp. 1, twenty-four multiparous sows (Landrace × Yorkshire; 90 d of gestation) were randomly assigned to two dietary treatment groups (*n* = 12) based on parity and historical reproductive performance including control group (CON) (sows received a basal diet containing 2% soybean oil) and BFAFP group (sows received a diet containing 2% BFAFP). The dietary composition of sows was provided in Supplementary Table S1, while the fatty acid profiles of soybean oil and BFAFP were detailed in Supplementary Table S2. The BFAFP (Shandong Crelipids Biotechnology Co., Ltd., Binzhou, China) was composed with deep-sea fish oil, flaxseed oil, red palm oil, soybean oil and lysophospholipids. The BFAFP had a U:S ratio of 2.98 and a n-6/n-3 PUFA ratio of 3.13 (Supplementary Table S2). Throughout the late gestation (from gestation day 90) and lactation periods (until weaning on 21 d), sows were individually housed in standard farrowing crates under controlled environmental conditions. Pregnant sows received ad libitum access to water and were provided measured feed allocations twice daily (07:00 h and 14:00 h), while lactating sows and their offspring had continuous access to both water and complete feed. To ensure exclusive maternal nutritional transfer, no supplemental milk replacers or creep feed were administered during the study period. Piglets were maintained with their respective dams and permitted unrestricted nursing access to facilitate natural suckling behavior and milk intake. On the day of parturition, 10 mL of venous blood was collected from the auricular vein of each sow. Blood samples were centrifuged and stored at −20 °C. Colostrum samples (10 mL) were collected from the 4th or 5th mammary glands after the birth of the fifth piglet and immediately frozen at −20 °C.

In Exp. 2, following 21-day lactation, twenty-four suckling piglets (Duroc × Landrace × Yorkshire; body weight 5.80 ± 0.10 kg; 21 days old) from these sows were selected and allocated to four groups (*n* = 6) in a 2 × 2 factorial design with the following factors: Maternal diet (soybean oil or BFAFP) and lipopolysaccharide (LPS) challenge (intraperitoneal injection of 100 μg/kg BW LPS or equivalent saline after a 12-h fast). At 4 h post-LPS challenge, piglets were euthanized for jejunal tissue and m jejunal mucosa collection. Deep anesthesia was first induced via intramuscular injection of Zoletil® 50 (10 mg/kg body weight). Subsequently, euthanasia was carried out by intravenous administration of an overdose of sodium pentobarbital (30 mg/kg body weight) through the ear vein. Death was confirmed by the absence of corneal reflex and the cessation of both heartbeat and respiration.

### 2.2 Reproductive performance of sows

Following parturition, reproductive performance metrics were systematically recorded for each sow, including farrowing characteristics (litter size, number of piglets born alive, and live farrowing rate), production parameters (farrowing duration and daily feed intake during lactation), lactation performance (milk yield and number of weaned piglets), and growth performance (total birth weight, weaning weight of individual piglet, total weaning weight, total weight gain at weaning, and daily gain of individual piglets). Milk yield = average daily weight gain of piglets (g/day) × 4 × number of weaned piglets × lactation days / (lactation days × 1,000) (17). All data were collected using standardized protocols to ensure consistency, with weaning piglet survival rate calculated as the percentage of live-born piglets surviving to weaning age.

### 2.3 Nutritional composition of colostrum

Colostrum samples were analyzed for nutritional composition using a fully automated fourier-transform infrared spectroscopy (FT-IR) milk analyzer (LactoScope 300, Delta Instruments, USA). Immunoglobulin concentrations (IgM and IgA) in sow colostrum were quantified using commercial ELISA kits (RX501029P and RX500977P; Quanzhou Ruixin Biotechnology Co., China) according to the manufacturer’s protocols.

### 2.4 Serum biochemical indexes

Triglycerides (TG), total cholesterol (TC), high density lipoprotein (HDL), and low density lipoprotein (LDL) of sow serum were measured by an Automatic Serum Biochemistry Analyzer (7,100, HITACHI, Tokyo, Japan) according to the standard protocols.

### 2.5 Disaccharidase activities

10-cm segments from mid-jejunum were opened longitudinally, rinsed with ice-cold PBS to remove contents, and the mucosa was collected by scraping with a glass slide. The mucosal samples were immediately snap-frozen in liquid nitrogen in RNase-free tubes and stored at −80 °C. The disaccharidase activities (lactase, sucrase, and maltase) in jejunum of piglets were determined using standardized assay kits (A082-1-1, A082-2-1, and A082-3-1; Nanjing Jiancheng, Nanjing, China) following the manufacturer’s instructions. All spectrophotometric measurements were performed in triplicate.

### 2.6 Intestinal morphology

Intestinal segments (3-cm) from mid-jejunum were rinsed with ice-cold PBS and fixed in 4% paraformaldehyde, subsequently dehydrated and embedded in paraffin using standard histological protocols. Sections (5 μm thickness) were prepared and stained with hematoxylin and eosin (H&E) for morphological evaluation according to Liu et al. (18). Villus height and crypt depth measurements were obtained from at least 10 intact, well-oriented crypt-villus units per sample at 100 × magnification.

### 2.7 Real-time quantitative PCR

Total RNA was extracted from piglet jejunum samples using RNAiso Plus (Takara, Japan) following the manufacturer’s instructions. RNA concentration and purity were determined spectrophotometrically using a NanoDro™ instrument, ensuring A260/A280 ratios were within the acceptable range of 1.8–2.1. Subsequently, cDNA was synthesized from total RNA via reverse transcription using a thermal cycler (PCR instrument). The mRNA expression levels of target genes were quantified using real-time quantitative PCR (qPCR). Relative gene expression was determined using the 2^−ΔΔCt^ method (19). Primer sequences used in this study are provided in Supplementary Table S3.

### 2.8 Western blotting analysis

Total protein was extracted from jejunum of piglets using the KGP2100 whole protein extraction kit (KeyGen Biotech, China) following the manufacturer’s protocol. Proteins were separated by SDS-PAGE before being transferred onto PVDF membranes (Millipore, USA). The membranes were then blocked and incubated overnight at 4 °C with primary antibodies: MFN1 (1:1000, Invitrogen, PA5-44826), MFN2 (1:1000, Abcam, ab56889), OPA1(1:1000, Novus, NB110-55290 s) and *β*-actin (1:1000, Sigma-Aldrich, A2228). Following washing with TBST, membranes were incubated with horseradish peroxidase (HRP)-conjugated secondary antibodies at room temperature. Protein bands were visualized using enhanced chemiluminescence (ECL) and quantified via densitometric analysis with GeneTools software (Syngene, Frederick, MD, USA).

### 2.9 Statistical analysis

All the data were analyzed using IBM SPSS statistical software (Version 22.0; IBM, Armonk, NY, USA). In Exp.1, data was analyzed by independent-sample T-test. In Exp. 2, data were analyzed by ANOVA using the general linear model procedures for a 2 × 2 factorial design. The model included the effects of LPS, BFAFP and their interaction. When significant BFAFP × LPS interactions occurred, multiple comparison tests were performed using Duncan’s multiple comparisons. All data were expressed as mean ± standard error. *p* ≤ 0.05 was considered statistically significant. Instances in which 0.05 < *p* ≤ 0.1 were considered as trends.

## 3 Results

### 3.1 The effects of maternal BFAFP supplementation on sow reproductive performance

Compared with CON group, sows receiving BFAFP supplementation demonstrated significant improvements in the number of weaned piglets (*p* < 0.05), weaning piglet survival rate (*p* < 0.05), and milk yield (*p* < 0.05) (Table 1). Notably, maternal BFAFP supplementation showed a tendency to increase total weaning weight per litter (*p* = 0.073) and total weight gain at weaning per litter (*p* = 0.058). Additionally, BFAFP supplementation tended to reduce farrowing duration (*p* = 0.051).

**Table 1:** Effects of dietary supplementation with BFAFP on sow reproductive performance.

Items	CON	BFAFP^1^	SEM	*p*
Litter size (per litter)	13.27	14.36	0.716	0.272
Number of piglets born alive (per litter)	13.00	13.64	0.620	0.512
Live farrowing rate (%)	98.13	98.82	1.471	0.770
Number of weaned piglets (per litter)	9.82^b^	11.70^a^	0.350	0.001
Weaning piglet survival rate (%)	90.56^b^	98.96^a^	2.180	0.020
Farrowing duration (h)	4.35	3.57	0.269	0.051
Daily feed intake during lactation (kg)	4.14	4.19	0.199	0.816
Milk yield (kg/d)^2^	8.01^b^	10.17^a^	0.479	0.021
Weaning weight of individual piglets (kg)	5.57	5.98	0.176	0.209
Total birth weight per litter (kg)	18.20	19.97	0.679	0.226
Total weaning weight per litter (kg)	57.23	69.81	3.190	0.073
Total weight gain at weaning per litter (kg)	42.06	52.02	2.640	0.058
Daily gain of individual piglets (g)	191	216	6.1	0.074

1 BFAFP: balanced fatty acids fat powder.

2 Milk yield = average daily weight gain of piglets (g/day) × 4 × number of weaned piglets × lactation days/(lactation days × 1,000).

Different small letters mean significant difference (*p* < 0.05).

### 3.2 The effects of maternal BFAFP supplementation on serum lipid metabolism indicators of sows

BFAFP supplementation significantly reduced plasma levels of TC, TG and HDL (*p* < 0.05) in sows (Table 2). BFAFP supplementation had a trend to decrease LDL level (*p* = 0.086) in sows.

**Table 2:** Effects of dietary supplementation with BFAFP on serum lipid metabolism indicators of sows.

Items	CON	BFAFP	SEM	*p*
TC (mmol/L)	2.01^a^	1.64^b^	0.144	0.019
TG (mmol/L)	0.23^a^	0.15^b^	0.028	0.006
HDL (g/L)	0.76^a^	0.62^b^	0.064	0.039
LDL (g/L)	0.81	0.68	0.075	0.086

Different letters mean significant difference (*p* < 0.05). BFAFP, balanced fatty acids fat powder; TG, Triglycerides; TC, total cholesterol; HDL, high density lipoprotein; LDL, low density lipoprotein.

### 3.3 The effects of maternal BFAFP supplementation on colostrum composition and immunoglobulin concentrations

**Figure 1:**
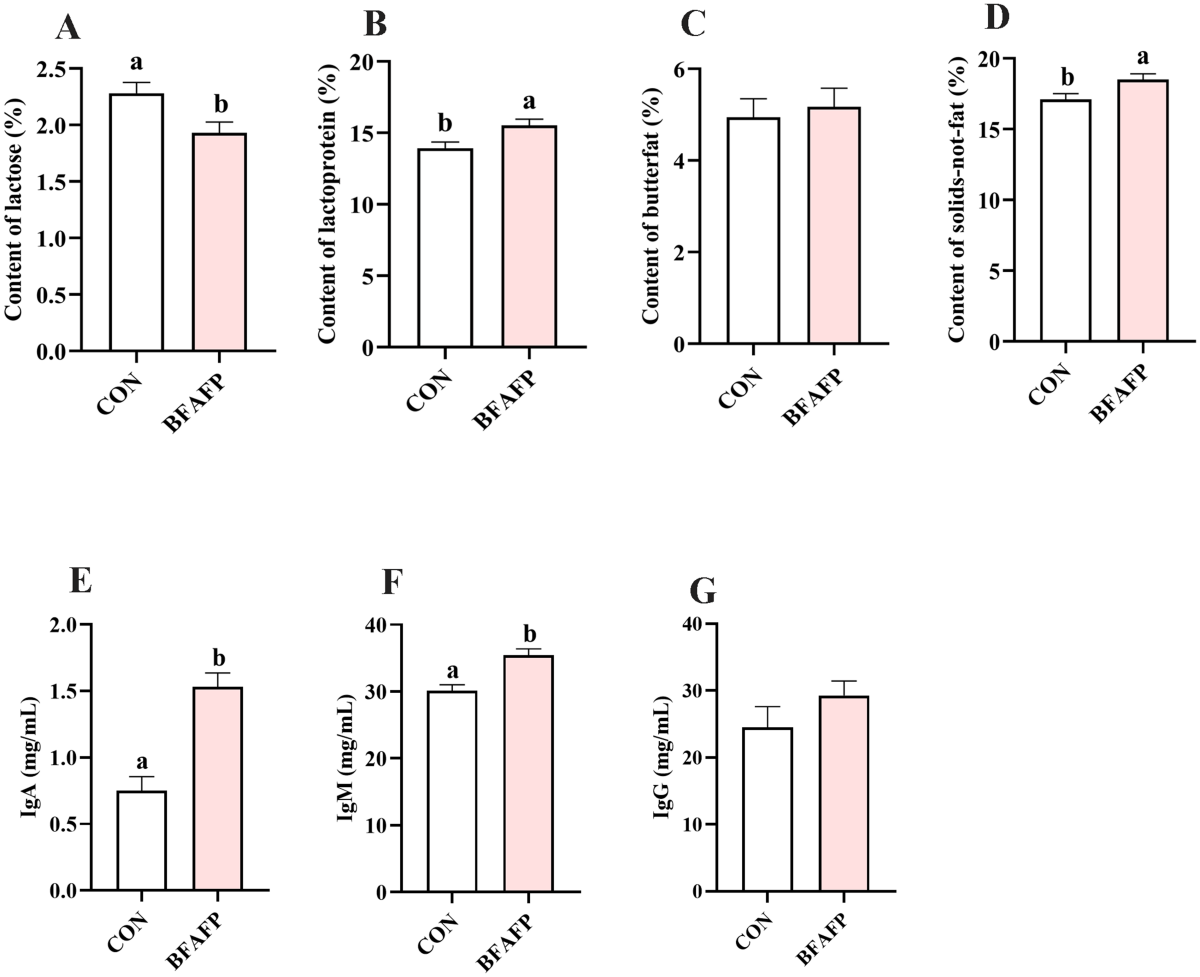
Effects of maternal BFAFP supplementation on colostrum composition and immunoglobulin concentrations. Sows in the CON group were fed a diet containing 2% soybean oil, whereas those in the BFAFP group received a diet supplemented with 2% BFAFP. **(A–D)** Concentrations of lactose, lactoprotein, butterfat, and solids-not-fat in colostrum. **(E–G)** Levels of IgA, IgM and IgG in colostrum. Data are presented as means ± SEM (*n* = 6). Bars labeled with different lowercase letters (a, b) indicate statistically significant differences (*p* < 0.05). BFAFP, balanced fatty acids fat powder; CON, control; IgA, immunoglobulin A; IgM, immunoglobulin M.

BFAFP supplementation significantly altered colostrum composition, with significant increases in lactoprotein (*p* < 0.05) and solids-not-fat content (*p* < 0.05), coupled with a significant reduction in lactose concentration (*p* < 0.01) compared to the CON group (Figures 1A–D). BFAFP significantly elevated concentrations of IgA (*p* < 0.05) and IgM (*p* < 0.05) relative to the CON group (Figures 1E,F). However, BFAFP did not affected the IgG level in colostrum (Figure 1G).

### 3.4 The effects of maternal BFAFP supplementation on intestinal morphology of piglets after LPS challenge

**Figure 2:**
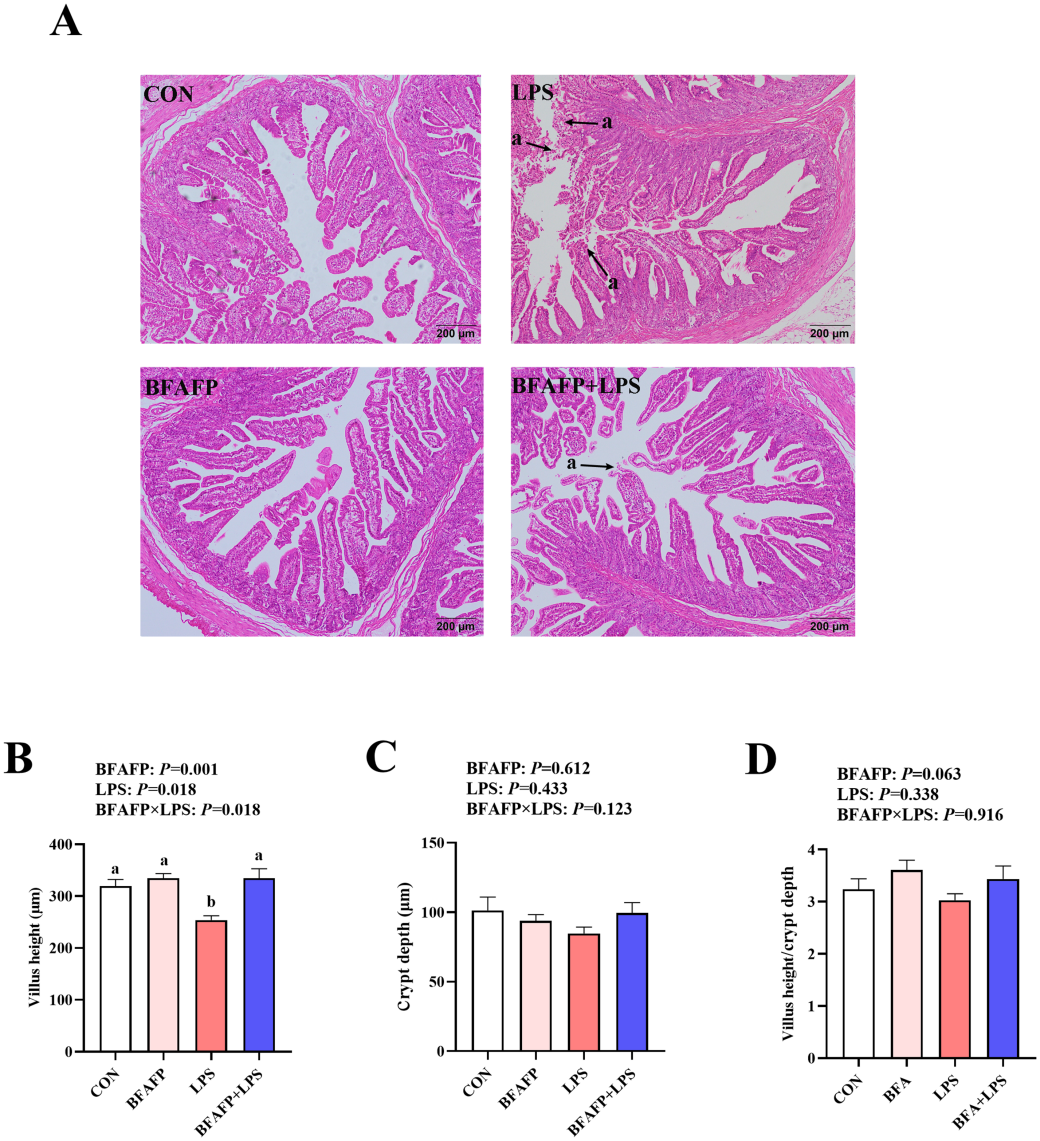
Effects of maternal BFAFP supplementation on intestinal morphology of suckling piglets following LPS challenge. Piglets were derived from sows fed either a 2% soybean oil diet (CON and LPS groups) or a 2% BFAFP-supplemented diet (BFAFP and BFAFP+LPS groups). Piglets in the CON and BFAFP groups received saline injections, whereas those in the LPS and BFAFP+LPS groups were challenged with LPS. **(A)** Representative hematoxylin and eosin (H&E)-stained jejunum sections; arrow indicates villus shedding. **(B–D)** Jejunal villus height, crypt depth, and villus height-to-crypt depth ratio. Data are expressed as means ± SEM (*n* = 6). Bars labeled with distinct lowercase letters (a, b) indicate significant differences (*p* < 0.05). BFAFP, balanced fatty acids fat powder; CON, control; H&E, hematoxylin and eosin; LPS, lipopolysaccharide.

LPS challenge significantly decreased villus height (*p* < 0.05) (Figure 2). A BFAFP×LPS interaction was found for villus height (*p* < 0.05) in which among LPS-injected piglets, maternal supplementation with BFAFP increased villus height, whereas the villus height did not differ among non-LPS-injected piglets.

### 3.5 The effects of maternal BFAFP supplementation on intestinal digestive function and barrier function of suckling piglets after LPS challenge

**Figure 3:**
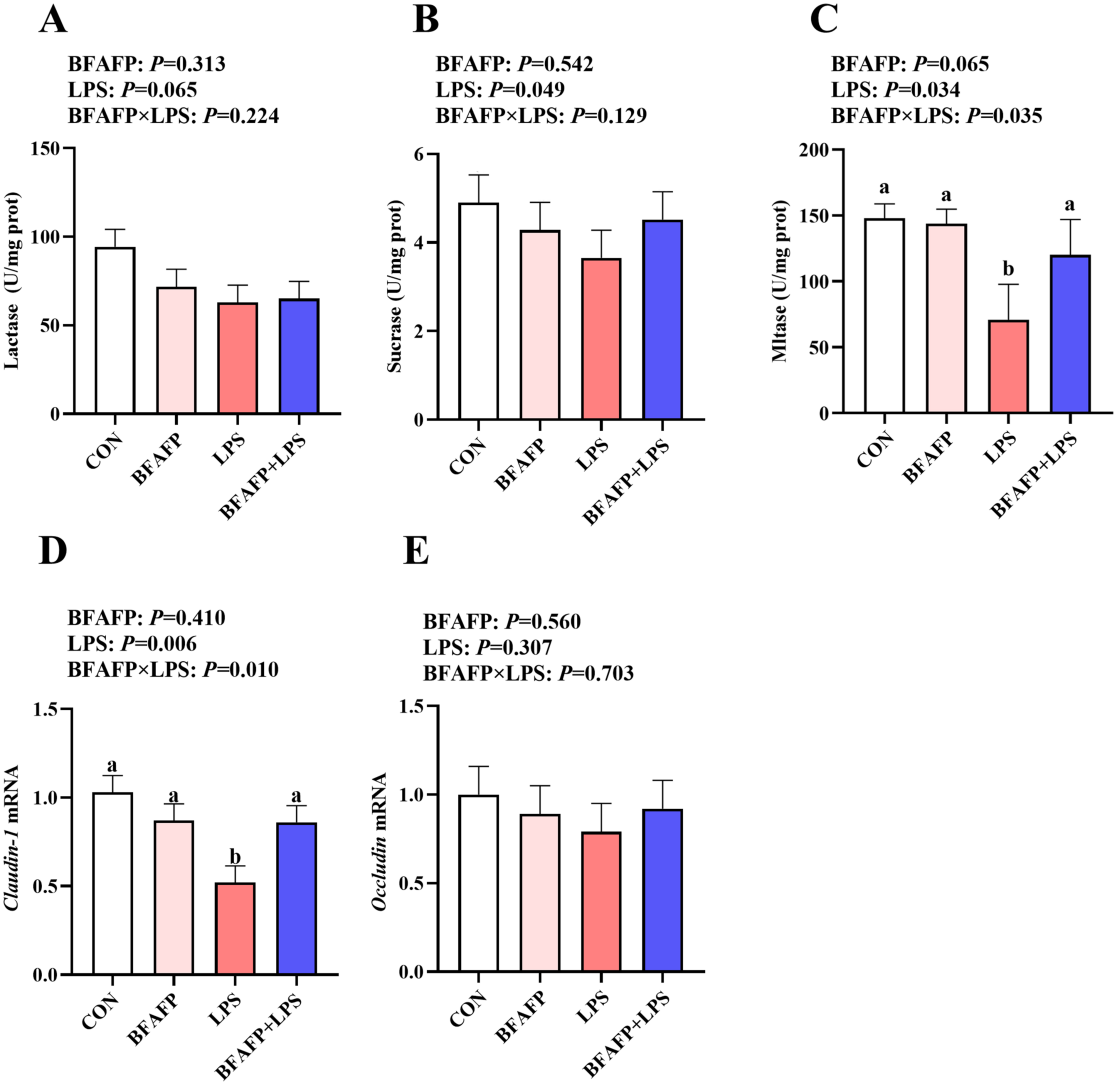
Effects of maternal BFAFP supplementation on intestinal digestive and barrier function in suckling piglets following LPS challenge. Piglets were derived from sows fed either a 2% soybean oil diet (CON and LPS groups) or a 2% BFAFP-supplemented diet (BFAFP and BFAFP+LPS groups). Piglets in the CON and BFAFP groups received saline injections, while those in the LPS and BFAFP+LPS groups were challenged with LPS. **(A–C)** Activities of lactase, sucrase, and maltase in the jejunum. **(D–E)** The mRNA abundance of tight junction proteins claudin-1 and occludin in jejunum. Data represent means ± SEM (*n* = 6). Different lowercase letters (a, b) indicate statistically significant differences (*p* < 0.05). BFAFP, balanced fatty acids fat powder; CON, control; LPS, lipopolysaccharide.

LPS challenge decreased maltase and sucrase activity in jejunum of piglets (*p* < 0.05) (Figures 3A–C), and had a trend to decrease lactase activity (*p* = 0.065). A BFAFP×LPS interaction was found for the maltase activity (*p* < 0.05) in which among LPS-injected piglets, maternal supplementation of BFAFP increased the maltase activity, whereas the maltase activity did not differ among non-LPS-injected piglets.

LPS challenge decreased mRNA expression of *claudin-1* in jejunum of piglets (*p* < 0.05) (Figures 3D–E). A BFAFP×LPS interaction was found for *claudin-1* mRNA (*p* < 0.05) in which among LPS-injected piglets, maternal supplementation with BFAFP increased mRNA abundance of *claudin-1*, whereas mRNA abundance of *claudin-1* did not differ among non-LPS-injected piglets. Neither LPS nor BFAFP had effects on jejunal *Occludin* mRNA expression.

### 3.6 The effects of maternal BFAFP supplementation on intestinal inflammation of suckling piglets after LPS challenge

**Figure 4:**
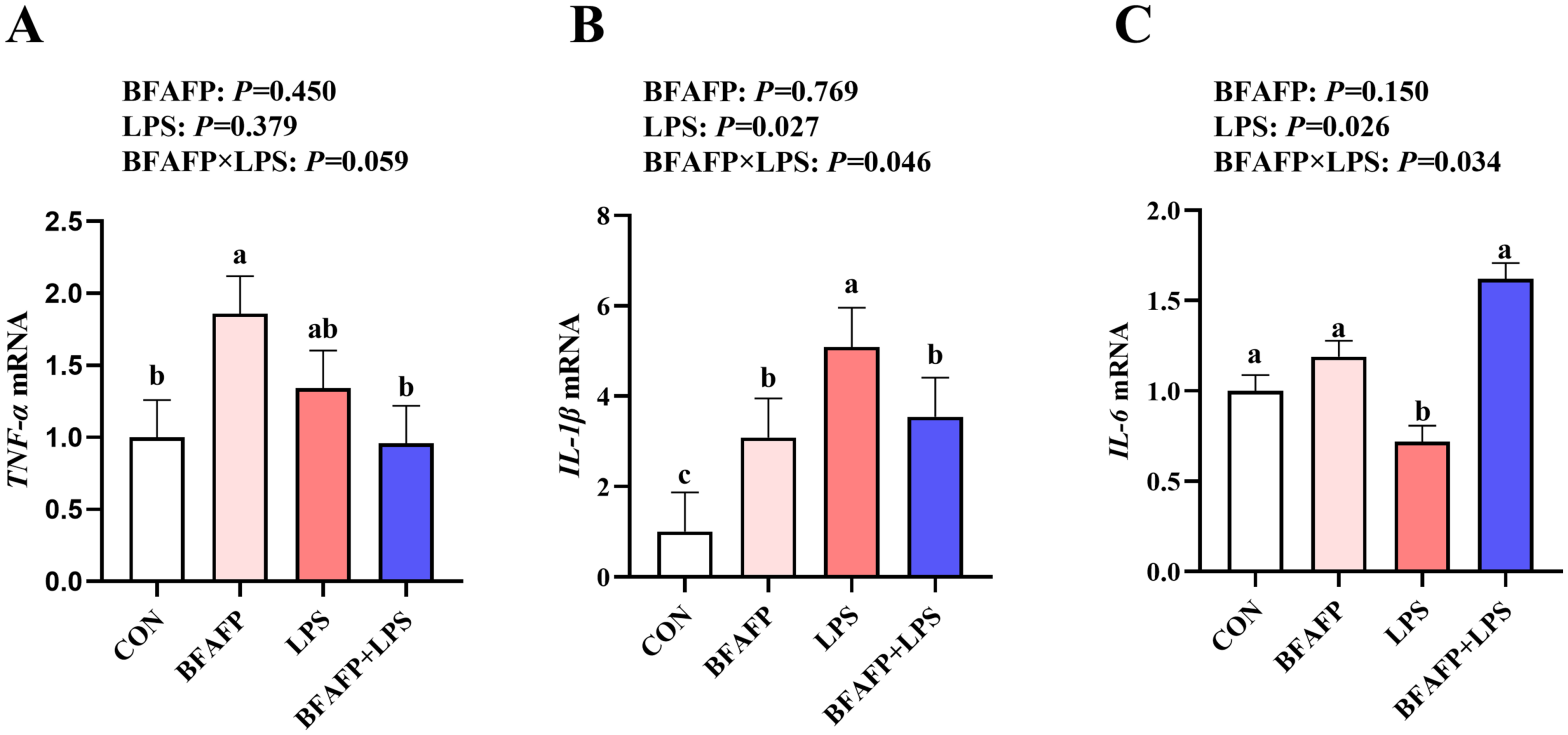
Effects of maternal BFAFP supplementation on jejunal inflammatory responses in suckling piglets following LPS challenge. Piglets were derived from sows fed either a 2% soybean oil diet (CON and LPS groups) or a 2% BFAFP-supplemented diet (BFAFP and BFAFP+LPS groups). All piglets received either saline (CON and BFAFP groups) or LPS (LPS and BFAFP+LPS groups) injections. **(A–C)** Relative mRNA expression levels of pro-inflammatory cytokines *TNF-α, IL-1β*, and *IL-6* in jejunum. Data are presented as mean ± SEM (*n* = 6). Different lowercase letters (a, b) indicate statistically significant differences (*p* < 0.05). BFAFP: balanced fatty acids fat powder; CON, control; LPS, lipopolysaccharide; *TNF-α*, tumor necrosis factor α; *IL-1β*, interleukin-1*β*; *IL-6*, interleukin-6.

LPS challenge significantly increased mRNA expression of *IL-1β* and *IL-6* in jejunum of piglets (*p* < 0.05) (Figure 4). Significant BFAFP × LPS interactions were found for mRNA expression of *IL-6* and *IL-1β* (*p* < 0.05). Among LPS-injected piglets, maternal supplementation with BFAFP increased mRNA expression of *IL-6,* whereas mRNA expression of *IL-6* did not differ among non-LPS-injected piglets. Maternal supplementation with BFAFP decreased mRNA expression of *IL-1β* in LPS-injected piglets, while BFAFP increased mRNA expression of *IL-1β* in non-LPS-injected piglets. A trend was observed for BFAFP × LPS interaction (*p* = 0.059) for *TNF-α* mRNA expression. While maternal BFAFP supplementation had no effect on *TNF-α* mRNA in LPS-injected piglets, it significantly increased its expression in non-LPS-injected piglets.

### 3.7 The effects of maternal BFAFP supplementation on intestinal mitochondria fusion of suckling piglets after LPS challenge

**Figure 5:**
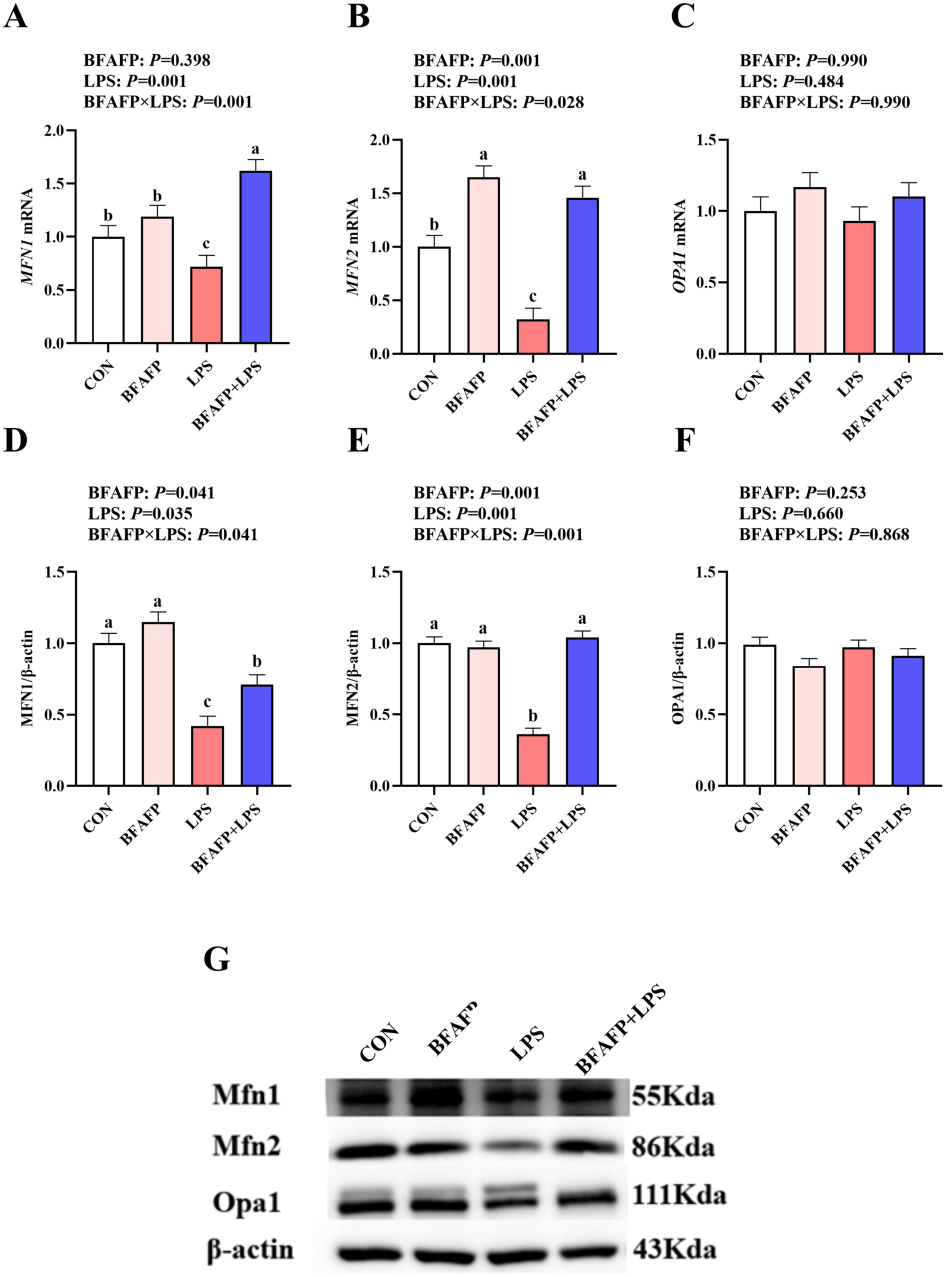
Effects of BFAFP supplementation on mitochondrial fusion protein expression in piglets following LPS challenge. Piglets were derived from sows fed either a 2% soybean oil diet (CON and LPS groups) or a 2% BFAFP-supplemented diet (BFAFP and BFAFP+LPS groups). Piglets received either saline (CON and BFAFP groups) or LPS (LPS and BFAFP+LPS groups) injections. **(A–C)** Relative mRNA expression levels of mitochondrial fusion genes (*MFN1*, *MFN2*, and *OPA1*) in jejunum. **(D–F)** Protein abundance of MFN1, MFN2, and OPA1 in jejunum. **(G)** Representative blots of MFN1, MFN2 and OPA1 proteins. Data represent mean ± SEM (*n* = 6). Different lowercase letters (a-d) indicate statistically significant differences (*p* < 0.05). BFAFP: balanced fatty acids fat powder; CON: control; LPS: lipopolysaccharide; MFN1: mitofusin1; MFN2: mitofusin2; OPA1: optic atrophy 1.

LPS challenge significantly decreased mRNA expression of *MFN1* and *MFN2* in jejunum (*p* < 0.05) (Figures 5A–C). BFAFP×LPS interactions were observed for mRNA expression of *MFN1* and *MFN2* (*p* < 0.05) in which among LPS-injected piglets, BFAFP supplementation increased mRNA expression of *MFN1* and *MFN2*, whereas mRNA expression of *MFN1* did not differ among non-LPS-injected piglets. Neither LPS or BFAFP had effects on mRNA expression of OPA1.

LPS challenge significantly decreased the abundance of MFN1 and MFN2 (*p* < 0.05) (Figures 5D–G). BFAFP×LPS interactions were observed for protein abundance of MFN1 and MFN2 (*p* < 0.05) in which among LPS-injected piglets, maternal BFAFP supplementation increased protein abundance of MFN1 and MFN2, whereas protein abundance of MFN1 and MFN2 did not differ among non-LPS-injected piglets.

### 3.8 The effects of maternal BFAFP supplementation on intestinal cell apoptosis of piglets after LPS challenge

**Figure 6:**
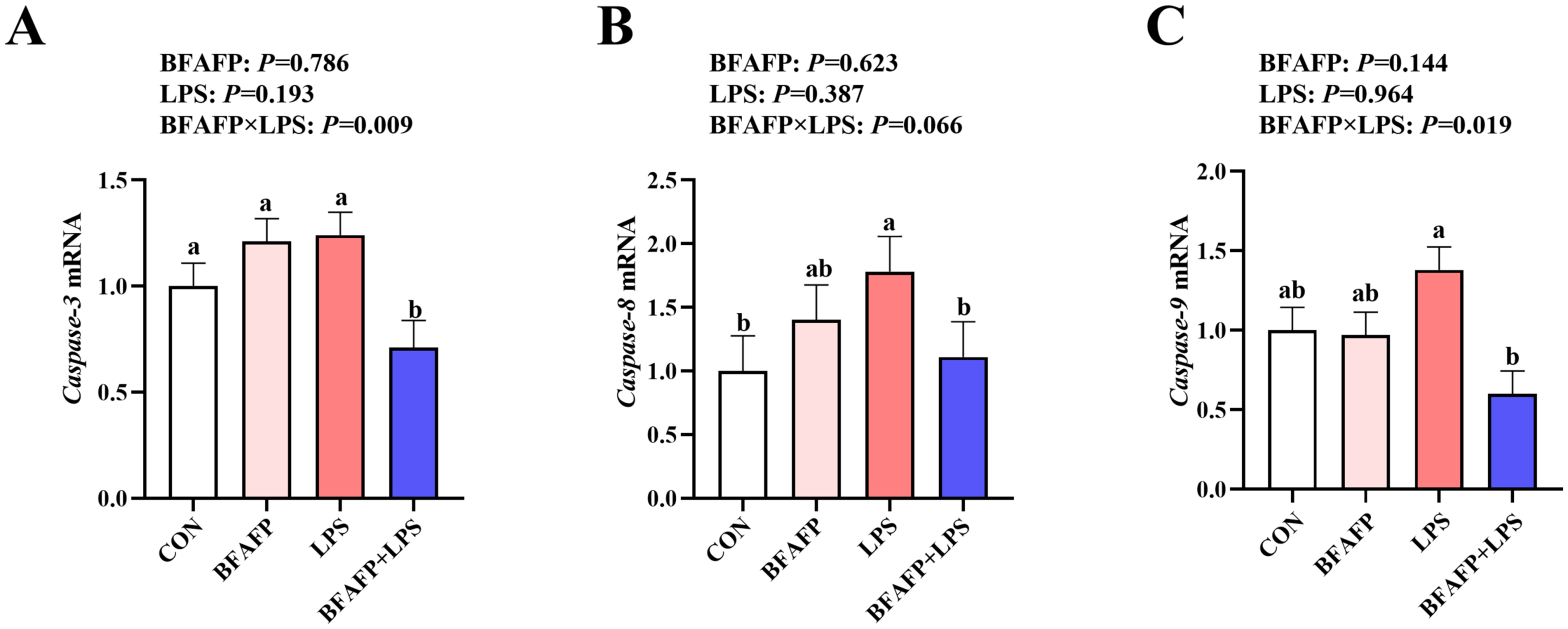
Effects of maternal BFAFP supplementation on apoptosis-related gene expression in jejunum of suckling piglets following LPS challenge. Piglets were derived from sows fed either a 2% soybean oil diet (CON and LPS groups) or a 2% BFAFP-supplemented diet (BFAFP and BFAFP+LPS groups), and subsequently administered either saline (CON and BFAFP groups) or LPS (LPS and BFAFP+LPS groups). **(A–C)** Relative mRNA expression levels of caspase-3, caspase-8, and caspase-9 in jejunum 4 h post injection. Data represent mean ± SEM (*n* = 6). Different lowercase letters (a–d) indicate statistically significant differences (*p* < 0.05). BFAFP, balanced fatty acids fat powder; CON, control; LPS, lipopolysaccharide.

BFAFP×LPS interactions were found for the mRNA expression of *caspase-3* and *caspase-9* (*p* < 0.05) in which among LPS-injected piglets, maternal supplementation with BFAFP decreased mRNA expression of *caspase-3* and *caspase-9*, whereas mRNA expression of *caspase-3* and *caspase-9* did not differ among non-LPS-injected piglets (Figure 6). Neither LPS or BFAFP had effects on mRNA expression of *caspase-8*. A trend for BFAFP×LPS interaction for *caspase-8* mRNA expression in which among LPS-injected piglets, maternal supplementation with BFAFP decreased mRNA expression of *caspase-8*, whereas mRNA expression of *caspase-8* did not differ among non-LPS-injected piglets.

## 4 Discussion

As suckling piglets rely solely on sows for nutrition, maternal dietary composition is critical for ensuring piglet intestinal health (2, 20). Fatty acids are particularly important for porcine growth and development, with varying requirements at different physiological stages (21, 22). Maintaining an appropriate fatty acids balance is essential for optimal porcine health and growth (23). This study investigated the effects of maternal appropriate n-6:n-3 PUFA and U:S ratio on sow reproductive performance and offspring growth and intestinal health, along with the underlying mechanisms.

In current study, our results indicated that maternal supplementation with BFAFP increased weaning piglet survival rate, the number of weaned piglets, and milk yield, and reduced farrowing duration, indicating that dietary BFAFP improved the reproductive performance of sows. This was similar with Nguyen et al. who reported that a low dietary n-6:n-3 ratio (4:1) during gestation and lactation was beneficial for weaning survival rate, and sucking piglets’ weight gain compared with high dietary n-6:n-3 ratio (10:1) (24). We speculated that BFAFP increased sow milk yield and quality, which directly led to a higher number of weaned piglets and improved weaning survival rate. Because high milk yield ensures an adequate and continuous supply of nutrition and high milk quality provides essential immunological and nutritional components (25). We also found that BFAFP supplementation decreased the TC, TG, HDL and LDL levels in serum of sows, suggesting that dietary BFAFP improved lipid metabolism in sows. This is supported by Sun et al. who showed that low n-6/n-3 PUFA ratio significantly reduced TG concentration in blood of participants (26). Chen et al. (27) also reported that the concentrations of serum TG and TC linearly increased with increasing of dietary n-6:n-3 PUFA ratio in finishing pigs. Regarding the mechanisms by which BFAFP reduced TC, TG, HDL, and LDL, we proposed that fish oil was likely the primary driver of TG reduction. It potently inhibited hepatic *de novo* lipogenesis while enhancing fatty acid *β*-oxidation via activation of the PPARα pathway (28). Additionally, lysophospholipids improved metabolic efficiency by enhancing lipid emulsification and absorption, which helps prevent postprandial lipid disturbances. Several plant-based oils contribute through their plant sterols and linoleic acid, which competitively inhibited intestinal cholesterol absorption. The combination of reduced input (absorption) and increased output (clearance) effectively lowered TC and LDL-C. Our results indicate that maternal supplementation with BFAFP can improve lipid clearance and decreased lipid absorption of sows.

Colostrum is a nutrient-dense biological fluid containing immunoglobulins and bioactive growth factors that are vital for postnatal development, immune protection, and survival during the critical transition from fetal to neonatal life (29). In our study, maternal BFAFP supplementation increased the contents of lactoprotein and non-fat solids in colostrum. Furthermore, it specifically elevated the levels of IgA and IgM, demonstrating a targeted enhancement of mucosal immunity via the gut-mammary axis (30). The increase in IgA and IgM strongly demonstrated that maternal BFAFP supplementation specifically enhanced the mucosal immunity and early humoral immunity of sows. The specific modulation, which did not extend to IgG content, suggested a refined immune response rather than a broad activation. This superior nutritional and immunological provision directly translated to improved offspring performance. Our findings align with previous research by Yao et al. who reported that sows fed a low n-6:n-3 PUFA ratio (3:1) exhibited higher colostral IgG and IgA contents compared to those fed a high ratio of n-6:n-3 PUFA (13:1) (31). This phenomenon may be attributed to the differential immunomodulatory effects of n-3 PUFA (anti-inflammatory) and n-6 PUFA (pro-inflammatory). Given that the BFAFP used in our study contained fish oil, flaxseed oil, and lysophospholipids, its composition likely contributed to the improvements in milk composition and immunoglobulin levels. The balanced n-3:n-6 PUFA ratio provided by these ingredients plays a crucial role in modulating immune responses. For example, fish oil supplementation significantly enhanced serum IgA concentration in piglets, with modest increases in IgM, while dietary adjustment of the n-3:n-6 ratio during the periparturient period significantly elevated plasma IgA and IgM in sows (32). Similarly, lysophospholipids have been demonstrated to enhance colostral IgA and IgM levels and improve milk components, an effect that acted in synergy with an optimized n-3:n-6 fatty acid ratio (33). Collectively, these results demonstrate that maternal BFAFP supplementation enhances the nutritional and immunological quality of colostrum, benefits that are closely associated with the regulation of the n-3:n-6 ratio, which may further confer advantages to piglet intestinal health.

The gastrointestinal tract serves as the primary site for nutrient digestion and absorption while simultaneously functioning as a critical physiological barrier against luminal pathogens, toxins, and other harmful agents (34). In suckling piglets, the intestinal system remains morphologically and functionally immature, rendering it particularly susceptible to exogenous and endogenous stressors during this critical developmental phase (35). In the present study, maternal supplementation with BFAFP attenuated LPS-induced intestinal damage in suckling piglets, as evidenced by improved jejunal villus architecture, enhanced epithelial cell proliferation, and increased activity of brush border enzymes such as maltase and sucrase. Furthermore, BFAFP supplementation upregulated claudin-1 mRNA expression following LPS challenge, suggesting a reinforcement of tight junction integrity and improved intestinal barrier function. While limited data exist on the effects of varying n-6:n-3 PUFA ratios and U:S ratios on porcine intestinal health, the observed benefits of BFAFP may be attributed to the primary lipid constituents of the supplemented fat powder. Supporting this notion, our previous studies demonstrated that both fish oil and flaxseed oil mitigated LPS-induced intestinal injury in weaned piglets (18, 36). We proposed that these benefits originated from the optimized fatty acid composition of BFAFP, specifically its balanced n-6/n-3 PUFA and U:S ratios. This composition reprogrammed the sow’s systemic metabolic environment (37). The altered maternal milieu was then translocated to the offspring transplacentally and via lactation, thereby priming the piglet’s immune system and intestinal epithelium for enhanced postnatal resilience. This priming effect, manifested as improved passive immunity or increased weaning weight due to greater milk yield, ultimately contributed to the pigs’ overall strength. These findings indicate that maternal BFAFP supplementation exerts protective effects on the intestinal structure and function of suckling piglets, highlighting its potential as a nutritional strategy to enhance gut health during early life stages.

Intestinal injury is usually associated with a marked increase in pro-inflammatory cytokine release (38). To assess the anti-inflammatory effects of BFAFP supplementation on offspring piglets, we analyzed the expression of key inflammatory mediators in jejunum of piglets. Our results demonstrated that maternal BFAFP supplementation significantly downregulated jejunal mRNA expression of *IL-1β* following LPS challenge, suggesting a potent suppressive effect on intestinal inflammatory responses. We propose that a lower n-6:n-3 PUFA ratio preferentially favored the incorporation of EPA and DHA into cell membranes over arachidonic acid. This compositional shift competitively altered eicosanoid precursor availability, thereby redirecting synthesis from pro-inflammatory arachidonic acid-derived mediators toward the less inflammatory and often pro-resolving mediators derived from EPA and DHA (39). Our findings agreed with previous studies highlighting the anti-inflammatory properties of dietary fatty acid modulation. Reddy et al. (40) reported that a balanced n-6:n-3 PUFA ratio in maternal diets attenuated colon inflammation in young adult rats. Similarly, McAfee et al. (41) observed that supplementing sows with protected fish oil during late gestation and lactation elevated the n-3:n-6 PUFA ratio in offspring and reduced systemic inflammatory cytokine levels post-weaning. Furthermore, existing evidence supports the anti-inflammatory efficacy of fish oil, flaxseed oil, and lysophospholipids in modulating intestinal immunity in swine (18, 36). Together, these data indicate that maternal BFAFP supplementation mitigates LPS-induced intestinal inflammation in suckling piglets, reinforcing its potential as a nutritional intervention to enhance gut health during early development.

Mitochondria, as dynamic energy-producing organelles, maintain cellular homeostasis through precisely regulated cycles of fission and fusion, processes essential for normal cellular function (42). This balance is particularly critical in the small intestinal epithelium, which undergoes complete self-renewal every 4–5 days and consequently exhibits exceptionally high energy demands (43). The mitochondrial fusion process is primarily mediated by three key proteins MFN1, MFN2, and OPA1 (15). Our investigation revealed that maternal supplementation with BFAFP significantly attenuated the LPS-induced downregulation of MFN1 and MFN2 expression in jejunum of suckling piglets. There was no data about the effects of balanced fatty acids in mitochondrial fusion. Our data firstly demonstrated that BFAFP played a protective role in maintaining mitochondrial fusion, therefore attenuating LPS-induced intestinal injury in suckling piglets. These data are supported by previous research that DHA supplementation enhanced immune cells mitochondrial fission and fusion in peripheral blood mononuclear cells from sportsmen (44), which suggesting a carry-over effect wherein the offspring’s mitochondrial function was primed by maternal nutrition. Specifically, the incorporation of maternally sourced n-3 PUFAs (e.g., DHA) into piglet tissues enhanced mitochondrial membrane fluidity (44). We proposed that this maternally programmed membrane environment facilitated Mfn1/2 dynamics, lowering the energy required for mitochondrial fusion in the offspring. N-3 PUFA have been shown to induce improvement in mitochondrial function and fusion processes associated with a reduction in reactive oxygen species production in both liver and skeletal muscle (45). Maternal fish oil supplementation reduced mitochondrial damage of offspring’s liver at weaning (46). These findings suggest that maternal BFAFP supplementation promotes mitochondrial fusion in the intestinal epithelium of offspring, potentially enhancing cellular energy metabolism and epithelial maintenance during critical developmental stages.

Imbalance in mitochondrial fission and fusion can trigger apoptotic pathways (42). Our results revealed that maternal supplementation with BFAFP significantly mitigated the LPS-induced upregulation of caspase-3 and caspase-9 mRNA expression. At present, the data about the ratio of n-6:n-3 PUFA and U:S on intestinal cell apoptosis is limited. Notably, the BFAFP formulation used in this study contained fish oil, which has been previously shown to exert anti-apoptotic and anti-inflammatory effects. For instance, fish oil pretreatment has been reported to attenuate cardiomyocyte apoptosis in mice, suppress proinflammatory cytokine production, and ameliorate sepsis-induced cardiomyopathy (47). Additionally, fish oil has demonstrated protective effects against ethanol-induced gastric damage by modulating apoptosis through nitric oxide-mediated pathways in humans (48). Moreover, n-3 PUFA (EPA and DHA) also showed an inhibitory effect on cell apoptosis in IPEC-1 cells after DON challenge (5, 49). We speculate that this maternally mediated carry-over effect operates through a mechanism wherein n-3 PUFAs from the maternal diet and their metabolites (including pro-resolving mediators) modulate offspring gene expression by influencing key transcription factors such as PPARγ and NF-κB (50). This transcriptional regulation promotes a shift in the balance of mitochondrial apoptosis regulators in the progeny, characterized by an increased Bcl-2/Bax ratio. The consequent enhancement of mitochondrial membrane stability thereby inhibits the permeability transition and the release of cytochrome c, a critical step in suppressing the apoptotic cascade (49, 50). Our findings support the conclusion that maternal BFAFP supplementation effectively suppresses LPS-induced apoptosis in the intestinal epithelial cells of suckling piglets.

In summary, maternal supplementation with BFAFP enhances sow reproductive performance and offspring growth performance, ameliorates LPS-induced intestinal structural and functional damage in piglets, promotes mitochondrial fusion, and suppresses intestinal epithelial cell apoptosis.

## Data Availability

The raw data supporting the conclusions of this article will be made available by the authors, without undue reservation.
