# Supplementary material for: Maternal supplementation with balanced fatty acid fat powder enhances sow reproductive performance and offspring intestinal health by modulating mitochondrial fusion and cell apoptosis
Source: Front Nutr. 2025 Dec 2;12:1690257. doi: 10.3389/fnut.2025.1690257 (PMC12705400; doi:10.3389/fnut.2025.1690257)
Supplement: Supplementary file 1 [file Table_1.docx]

**Table S1** Dietary composition and nutritional levels of sows

| Ingredients | Soybean oil group (%) | BFAFP^a^ group (%) |  |
| --- | --- | --- | --- |
| Corn | 51.80 | 51.80 |  |
| Soybean meal (43%) | 16.50 | 16.50 |  |
| Bran (17%) | 9.00 | 9.00 |  |
| Wheat (13.9%) | 6.80 | 6.80 |  |
| Super steamed fish meal (67%) | 2.00 | 2.00 |  |
| Dried porcine soluble (50%) | 1.25 | 1.25 |  |
| Expanded soybean (35%) | 5.00 | 5.00 |  |
| Soybean oil | 2.00 | 0.00 |  |
| BFAFP^a^ | 0.00 | 2.00 |  |
| Sugar | 1.00 | 1.00 |  |
| Limestone | 1.00 | 1.00 |  |
| Calcium hydrogen phosphate | 1.70 | 0.7 |  |
| Premix^b^ | 1.00 | 1.00 |  |
| Total | 100.00 | 100.00 |  |
| Nutritional levels^c^ |  |  |  |
| Digestible energy (MJ/kg) | 13.85 | 13.63 |  |
| Crude protein (%) | 18.32 | 18.32 |  |
| Crude fat (%) | 3.50 | 3.50 |  |
| Calcium (%) | 1.05 | 1.05 |  |
| Total phosphorus (%) | 0.87 | 0.87 |  |
| SID Lysine (%) | 0.91 | 0.91 |  |
| SID Methionine (%) | 0.45 | 0.45 |  |
| SID Threonine (%) | 0.76 | 0.76 |  |
| SID Tryptophan (%) | 1.16 | 1.16 |  |
| SID Valine (%) | 0.87 | 0.87 |  |

^a^BFAFP: balanced fatty acids fat powder.

^b^Provided per kilogram of diet: vitamin A, 9,000 IU; vitamin E, 120 IU; vitamin D_3_, 2500 IU; vitamin B_1_, 3 mg; vitamin B_2_, 8 mg; vitamin B_12_, 0.03 mg; niacin, 60 mg; pantothenic acid, 25 mg; folic acid, 2.5mg; biotin, 0.3mg; Cu (CuSO_4_·5H_2_O), 30 mg; Fe (FeSO_4_·H_2_O), 80 mg; Zn (ZnSO_4_·7H_2_O), 90 mg; Mn (MnSO_4_·5H_2_O), 45 mg; Se (Na_2_SeO_3_), 0.3 mg.

^c^Nutritional levels were calculated values.

**Table S2** Fatty acid composition of soybean oil and BFAFP.

| Items | Soybean oil | | BFAFP^1^ |
| --- | --- | --- | --- |
|  | % total fatty acids | | |
| C8:0 | 0.01 | 0 | |
| C10:0 | 0.01 | 0 | |
| C12:0 | 0.01 | 0.15 | |
| C14:0 | 0.08 | 1.15 | |
| C15:0 | 0.02 | 0 | |
| C16:0 | 10.72 | 19.80 | |
| C16:1(n-7) | 0.09 | 1.09 | |
| C17:0 | 0.10 | 0 | |
| C18:0 | 4.43 | 3.85 | |
| C18:1(n-9) | 22.05 | 23.93 | |
| C18:2(n-6) | 54.17 | 37.76 | |
| C18:3 (n-3) | 7.02 | 8.62 | |
| C20:0 | 0.39 | 0.18 | |
| C20:1(n-7) | 0.20 | 0 | |
| C20:4(n-6) | 0 | 0.72 | |
| C20:5(n-3) | 0 | 1.16 | |
| C21:0 | 0.05 | 0 | |
| C22:0 | 0.43 | 0 | |
| C22:1(n-9) | 0.02 | 0 | |
| C22:6(n-3) | 0 | 1.59 | |
| C23:0 | 0.06 | 0 | |
| C24:0 | 0.15 | 0 | |
| U:S | 5.08 | 2.98 | |
| n-6/n-3 | 7.72 | 3.13 | |

BFAFP: balanced fatty acids fat powder. Product is provided by Shandong Crelipids Biotechnology Co., Ltd.

**Table S3** Primers used in real-time quantitative PCR

| Gene | Accession | Direction | Sequences (5’-3’) | | Size |
| --- | --- | --- | --- | --- | --- |
| *IL-1β* | NM_214055.1 | Forward | F：GCTAACTACGGTGACAACAATAATG | | 186 |
|  |  | Reverse | R：CTTCTCCACTGCCACGATGA | |  |
| *IL-6* | NM_214403.1 | Forward | F：CTCCAAACTGGAGGTGGCG |  | 114 |
|  |  | Reverse | R：CTGAGCACCCAGTGAATGGT |  |  |
| *TNF-α* | NM_214022 | Forward | F：TCCAATGGCAGAGTGGGTATG |  | 67 |
|  |  | Reverse | R：AGCTGGTTGTCTTTCAGCTTCAC |  |  |
| *Claudin-1* | NM_001244539.1 | Forward | F：AGATTTACTCCTACGCTGGT |  | 249 |
|  |  | Reverse | R：GCACCTCATCATCTTCCAT |  |  |
| *Occludin* | NM_001163647.1 | Forward | F：ATCAACAAAGGCAACTCT |  | 157 |
|  |  | Reverse | R：GCAGCAGCCATGTACTCT |  |  |
| *Caspase-3* | NM_214131.1 | Forward | F：ACCCAAACTTTTCATAATTCA |  | 145 |
|  |  | Reverse | R：ACCAGGTGCTGTAGAATATGC |  |  |
| *Caspase-8* | NM_001031779.2 | Forward | F：ACAACTACATCCTCCTAACG | | 293 |
|  |  | Reverse | R：CTCTCCATCTCCTCCTCAT | |  |
| *Caspase-9* | XM_013998997.2 | Forward | F：AACCAGTAGACAAGCAACAA | | 209 |
|  |  | Reverse | R：TGAATCCTCCAGAACCAATG | |  |
| *GAPDH* | AF017079.1 | Forward | F：CGTCCCTGAGACACGATGGT | | 194 |
|  |  | Reverse | R：GCCTTGACTGTGCCGTGGAAT | |  |

IL-1β: interleukin-1 β; IL-6: interleukin-6; TNF-α: tumor necrosis factor α; GAPDH: glyceraldehyde-3-phosphate dehydrogenase.
